# Approaches Towards Professional Studies and Spare-time Activities Among Preclinical and Clinical Year Medical Students

**DOI:** 10.7759/cureus.4905

**Published:** 2019-06-14

**Authors:** Tahir Jameel, Mukhtiar Baig, Zohair J Gazzaz, Jawad M Tashkandi, Nasser S Al Alhareth, Shahida A Khan, Nadeem S Butt

**Affiliations:** 1 Internal Medicine, King Abdulaziz University, Jeddah, SAU; 2 Clinical Biochemistry, Medical Education, Faculty of Medicine, King Abdulaziz University, Jeddah, SAU; 3 Miscellaneous, Faculty of Medicine, King Abdulaziz University, Jeddah, SAU; 4 Community Medicine, King Fahad Medical Research Center, King Abdulaziz University, Jeddah, SAU; 5 Medical Statistics, Faculty of Medicine, King Abdulaziz University, Jeddah, SAU

**Keywords:** medical students, study habits, reading resources, social media, clinical sciences, basic sciences

## Abstract

Objective

This study aims at a recognition of the differences in the study habits, approach to teaching resources, and spare-time activities of medical students in the preclinical and clinical training periods at King Abdulaziz University (KAU) Jeddah, Saudi Arabia (SA).

Methods

Study sampling was carried out in 2017 at the Faculty of Medicine, KAU, Jeddah, SA. Students from both genders were included and further subdivided to preclinical (2^nd^ and 3^rd^ years) and clinical groups (4^th^, 5^th^, and 6^th^ years). Students were asked to respond to an online questionnaire. SPSS-Version 21 (IBM Corp., Armonk, NY, US) was utilized for statistical analysis of the collected data,

Results

Of the 347/500 (response rate 69.4%) medical students, 85 (24.5%) were from the preclinical students (2^nd^ and 3^rd^ years), and 262 (64.5%) were enrolled in the clinical group (4^th^ to 6^th^ years of MBBS). The majority of students 330 (94.1%) were unmarried, only 17 of them, i.e., 4.9%, were married. Analysis of the data revealed that medical textbooks, essential versions of basic medical books, online resources, and online version of books were used more frequently by the clinical group as compared to the preclinical students. Teacher-provided lecture handouts and lecture notes taken during classes were being equally used by both groups. There was a significant difference in the opinion on the usefulness of different resources between both groups.

Students faced difficulty in understanding the English language, observed more in the pre-clinical years as compared to relatively groomed clinical students. The preclinical group could not understand the teaching material in books due to a weaker understanding of the English language. Social media software was used for keeping both groups busy, but clinical students also used social media for academic purposes. More than half of the participants from the preclinical and almost one-third from the clinical years admitted that their teachers recommended them for relevant medical textbooks. An encouraging trend was observed in most preclinical group students: they found teaching modalities, such as problem-based learning (PBL) and other academic activities, as a trigger to promote book reading.

Conclusion

Our results show that the students in the clinical phase had a more methodical approach to professional studies and a difference in spare-time activities.

## Introduction

Medical education is one of the toughest fields and needs plenty of time, dedication, passion, and concentration. Mastery of the subject depends upon the approach that the students adopt for learning. One of the most common complaints among medical students is the excessive burden of the curriculum and frequent assessments [1]. Acquiring knowledge and essential clinical skills are fundamental for the grooming of the developing health professional. Reading is the most crucial way of learning throughout the medical carrier [2]. It all depends on how efficiently a medical student has mastered his/herself in time management, concentration, reading speed, study habits, and retaining knowledge by proper revision sessions [3].

Medical education traditionally involves training activities, which prepare a young, raw individual in becoming a member of the noble society of humans [3]. It involves biphasic training, i.e., preclinical and clinical. Preclinical training grooms individuals with background knowledge, which remains the backbone of one’s critical decisions throughout the professional career [4-5]. Preclinical or basic sciences teaching, including anatomy, physiology, pharmacology, biochemistry, and other subjects, are said to have three basic responsibilities. First, to provide the backbone for future clinical reasoning; second, to facilitate management decisions; these may be of a medical or surgical nature; and third, to support plus encourage improvements in tricky healthcare situations [6].

The lengthy curriculum and frequent assessment also influence the students’ approach to learning and studying habits and spare-time activities. A study demonstrated a significant relationship between study habits and performance in professional assessments [7]. The abundance of knowledge in the field of medical sciences compels medical students to adopt different learning approaches for passing the exams, and this is significantly influenced by the way of assessment as well [8]. The learning requirements of a medical student are partly different at various stages of professional study. In most medical schools, the standard practice of medical teaching is aligned in such a way that in preclinical years, the students usually learn in the classrooms, with occasional introductory visits to the hospitals, while during the training in clinical years, the main focus is on bedside teaching [9]. Students acquire clinical skills while being part of multidisciplinary healthcare teams in hospitals. It has been observed that females are more attentive and time-sparing as compared to boys in preclinical years when most of the information is provided through textbooks and related methods [10]. A recent study reported that female medical students study books more frequently as compared to males [11]. The grades of preclinical students mostly depend on how much time they spare for reading and retaining knowledge [12].

Medical students face several academic stressors like lengthy academic curriculum or syllabus, frequent periodic exams, theoretical and practical exams, and a lack of time for other activities [13]. To overcome these stresses, medical students adopt different approaches to cover the extensive curriculum and pass frequent exams. There is a lack of published data regarding the comparison of the medical students' approaches in the preclinical and clinical training years towards professional studies in SA. Only a few studies explored this topic in the region [14-15]. This study aims at a recognition of the differences in study habits, approach to teaching resources, and spare-time activities of medical students in the preclinical and clinical training period in King Abdulaziz University (KAU), Jeddah, Saudi Arabia.

## Materials and methods

Study sampling was carried out over three months in 2017 at King Abdulaziz University, Jeddah. The study was conducted on two campuses, i.e., Faculty of Medicine, Rabigh, and KAU Jeddah. The preclinical group comprised 2^nd^ and 3rd-year students, and 4^th^, 5^th^, and 6th-year students were part of the “clinical” group. In Saudi Arabia, medical students spend their first year (foundation year) in training in the English language, chemistry, and biology, along with other basic premedical subjects. The students belonged to the Faculty of Medicine, Rabigh and Jeddah campuses of the KAU. The Rabigh campus is a comparatively newly established campus of KAU and situated 150 km away from Jeddah city. The participants were briefed in detail regarding the aim of the study, and verbal consent was obtained from all the participants. The Ethical Committee of King Abdulaziz University Jeddah granted research approval.

All the students had access to an online questionnaire, having detailed queries regarding the evaluation of the students’ approaches towards professional studies and how their spare time was being utilized in different hobbies and activities. The questions were constructed according to the Likert scale. Multiple-choice questions were also included so that a wide choice could be available to the students for their responses. The questionnaire was presented to around 500 medical students, both males and females, of all the classes of MBBS. Initially, the questionnaire was tested on a pilot group of 30 students, and Cronbach alpha was calculated (Cronbach alpha=0.81). Our consultant team consisted of a medical educationist and a senior professor who confirmed the content validity. We modified our questionnaire according to the suggestions of our experts.

SPSS, Version 21 (IBM Corp., Armonk, NY, US) was utilized for statistical analysis and variables were expressed as frequencies and percentages. The Chi-square test was used to find out significant differences among different variables and p-value <.05 was taken as significant.

## Results

Of the 347/500 (response rate 69.4%) medical students, 85 (24.5%) were from the preclinical students (2^nd^ and 3^rd^ years), and 262 (64.5%) were enrolled in the clinical group (4^th^ to 6^_th_^ year of MBBS). Most students, 330 (94.1%), were unmarried; only 17 of them, i.e., 4.9%, were married.

The clinical group, as compared to the preclinical group, used medical books, essential versions of basic medical books, online resources, and online version of books more frequently. Teacher-provided lecture handouts and lecture notes taken during classes were being equally used by both groups (Table 1).

**Table 1:** Comparison of time spent/day by the preclinical and clinical students in using different resources (students were allowed to tick more than one item) *. The Chi-square statistic is significant at the .05 level.

		Level of Study				P-Value
		Pre-Clinical		Clinical		
		n	%	n	%	
Medical textbook	Rarely	45	45.0%	55	55.0%	<0.001*
	<30 Mins/day	13	19.4%	54	80.6%	
	30-60 Mins/day	10	12.2%	72	87.8%	
	60-90 Mins/day	8	18.6%	35	81.4%	
	>90 Mins/day	9	16.4%	46	83.6%	
Essential version of a basic medical text book	Rarely	46	33.8%	90	66.2%	0.006*
	<30 Mins/day	25	24.0%	79	76.0%	
	30-60 Mins/day	7	13.0%	47	87.0%	
	60-90 Mins/day	4	17.4%	19	82.6%	
	>90 Mins/day	3	10.0%	27	90.0%	
Online sources:	Rarely	25	33.3%	50	66.7%	0.018*
	<30 Mins/day	34	26.8%	93	73.2%	
	30-60 Mins/day	18	20.0%	72	80.0%	
	60-90 Mins/day	1	3.3%	29	96.7%	
	>90 Mins/day	7	28.0%	18	72.0%	
Online version of textbook	Rarely	44	30.8%	99	69.2%	0.035*
	<30 Mins/day	24	25.0%	72	75.0%	
	30-60 Mins/day	10	16.7%	50	83.3%	
	60-90 Mins/day	7	21.9%	25	78.1%	
	>90 Mins/day	0	0.0%	16	100.0%	
Medical websites	Rarely	26	32.1%	55	67.9%	0.005
	<30 Mins/day	39	27.7%	102	72.3%	
	30-60 Mins/day	12	18.2%	54	81.8%	
	60-90 Mins/day	6	16.2%	31	83.8%	
	>90 Mins/day	2	9.1%	20	90.9%	
Pocketbooks	Rarely	58	32.2%	122	67.8%	0.005*
	<30 Mins/day	14	16.7%	70	83.3%	
	30-60 Mins/day	10	22.2%	35	77.8%	
	60-90 Mins/day	3	11.5%	23	88.5%	
	>90 Mins/day	0	0.0%	12	100.0%	
Lecture handouts	Rarely	8	21.1%	30	78.9%	0.491
	<30 Mins/day	20	27.4%	53	72.6%	
	30-60 Mins/day	18	18.6%	79	81.4%	
	60-90 Mins/day	20	27.4%	53	72.6%	
	>90 Mins/day	19	28.8%	47	71.2%	
Lecture notes taken in the class	Rarely	13	20.6%	50	79.4%	0.182
	<30 Mins/day	29	20.7%	111	79.3%	
	30-60 Mins/day	17	24.3%	53	75.7%	
	60-90 Mins/day	14	36.8%	24	63.2%	
	>90 Mins/day	12	33.3%	24	66.7%	

There was a significant difference in the opinion regarding the usefulness of different resources between both groups (Table 2).

**Table 2:** Comparison of the preclinical and clinical students’ perception regarding usefulness of different resources (students could tick more than one item) *. The Chi-square statistic is significant at the .05 level.

		Level of Study				P-value
		Pre-Clinical		Clinical		
		Count	Row N %	Count	Row N %	
Medical textbook	Not useful at all	16	47.1%	18	52.9%	<0.001
	Somewhat useful	48	31.4%	105	68.6%	
	Extremely useful	21	13.1%	139	86.9%	
Essential version of a basic medical text book	Not useful at all	25	48.1%	27	51.9%	<0.001
	Somewhat useful	42	23.9%	134	76.1%	
	Extremely useful	18	15.1%	101	84.9%	
Online sources	Not useful at all	11	39.3%	17	60.7%	0.018
	Somewhat useful	40	29.0%	98	71.0%	
	Extremely useful	34	18.8%	147	81.2%	
Online version of textbook	Not useful at all	20	30.8%	45	69.2%	0.003
	Somewhat useful	49	29.9%	115	70.1%	
	Extremely useful	16	13.6%	102	86.4%	
Online journal article	Not useful at all	34	37.0%	58	63.0%	0.002
	Somewhat useful	43	22.2%	151	77.8%	
	Extremely useful	8	13.1%	53	86.9%	
Medical websites	Not useful at all	16	51.6%	15	48.4%	<0.001
	Somewhat useful	40	29.2%	97	70.8%	
	Extremely useful	29	16.2%	150	83.8%	
Pocketbooks	Not useful at all	37	50.0%	37	50.0%	0.000
	Somewhat useful	38	22.4%	132	77.6%	
	Extremely useful	10	9.7%	93	90.3%	
Journal articles (print version	Not useful at all	36	27.7%	94	72.3%	0.522
	Somewhat useful	42	23.1%	140	76.9%	
	Extremely useful	7	20.0%	28	80.0%	
Lecture handouts	Not useful at all	10	35.7%	18	64.3%	0.004
	Somewhat useful	23	15.6%	124	84.4%	
	Extremely useful	52	30.2%	120	69.8%	
Test Preparation textbooks	Not useful at all	18	34.6%	34	65.4%	0.170
	Somewhat useful	36	21.8%	129	78.2%	
	Extremely useful	31	23.8%	99	76.2%	
Lecture notes taken in the class	Not useful at all	6	24.0%	19	76.0%	0.142
	Somewhat useful	23	18.5%	101	81.5%	
	Extremely useful	56	28.3%	142	71.7%	

Preclinical students faced difficulty in understanding the English language; they admitted that because of this problem, they did not readily understand the professional language of books. The trend of avoiding textbooks was observed more in preclinical students as compared to students in clinical years, as they must depend more on medical literature in dealing with different clinical challenges as compared to lecture notes by the teachers. The preclinical students had many other excuses to avoid textbook reading like feeling it is difficult to focus on a relevant topic, not finding enough time to go through the voluminous books, or facing difficulty in finding appropriate resources according to their specific needs (Table 3).

**Table 3:** Comparison of the preclinical and clinical students experience regarding reading the textbooks *. The Chi-square statistic is significant at the .05 level.

		Level of Study				P-value
		Pre-Clinical		Clinical		
		n	%	n	%	
I don’t like reading material written in English	Agree	53	62.3	15	5.7	<0.000
	disagree	23	27.0	243	92.7	
	Don’t know	9	10.5	4	1.5	
I have problem in understanding the textbook	Agree	39	45.8	20	7.6	<0.000
	disagree	30	35.2	242	92.3	
	Don’t know	16	18.8	--	---	
I don’t have enough time to read textbook	Agree	56	65,8	16	6.1	<0.000
	disagree	15	17.6	246	93.8	
	Don’t know	14	16.4	--	--	
I don’t know what to focus in reading textbook	Agree	60	70,5	7	2.6	<0.000
	disagree	25	29.5	237	90.4	
	Don’t know	--	--	18	6.8.0	
I don’t know the best resources	Agree	25	29.4	10	3,8	<0.000
	disagree	55	64,7	245	93.5	
	Don’t know	1	1.17	7	2.6	
I feel difficulty to find sources appropriate to my level	Agree	41	48.2	12	4.5	<0.000
	disagree	38	44.7	235	89.6	
	Don’t know	6	7.0	15	5.7	

**Figure 1:**
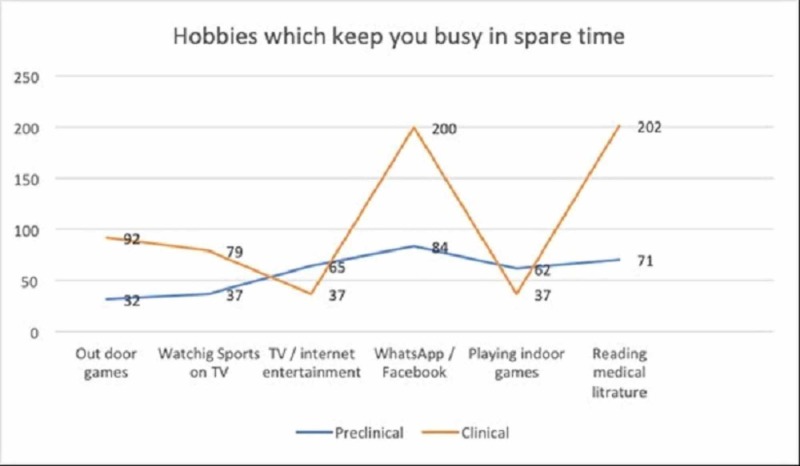
Recreational activities among preclinical and clinical years medical students

Most clinical year students 202 (77.0%) committed to spending most of their spare time while studying the medical literature. Similar trends were observed in response to questions about outdoor games and watching sports on TV. The use of social media software was equally popular between the groups. Almost 98% of preclinical and 76% of clinical students mentioned WhatsApp and Facebook as their major way of passing time (Figure 1).

More than half of the participants, i.e., 52 (61%), of the preclinical and 99 (38%) from the clinical year admitted that their teachers recommended them for relevant medical textbooks. Significantly, many students of the preclinical group, 82 (92%), thought that problem-based learning (PBL) and other active learning activities promoted the student’s interest in book reading to a great extent. However, only a small group of clinical students also mentioned it as a useful teaching tool. As the preclinical students consulted the library relatively quite often, they appreciated that all the books in the college library were updated regularly and, mostly, the textbooks were of the latest edition.

## Discussion

In the clinical phase, the students are exposed to new clinical queries because they are more inclined towards online journals and medical websites [16]. A similar trend was seen in our study where 91% of our clinical students preferred medical websites along with other online sources. Interestingly, the clinical students in our cohort were given enough time to use medical textbooks as compared to the preclinical students. Similarly, it has been observed by Kauffman (2015) that because of the change in learning climate after exposure to patients, the students in the clinical phase give importance to the latest editions of the textbooks and to recent information provided by medical websites and online journal articles [17].

Mon et al. (2014) pointed out the preference of preclinical medical students towards the subject related textbooks, lecture handouts, and test preparation books. The primary purpose being the success in assessment tasks frequently posed to preclinical students [18]. However, we observed a relatively different trend in our study that the clinical students outnumbered the preclinical group in the study of almost all the study resources. It was statistically highly significant while comparing both groups on medical book reading, essential version of the books, pocketbooks, medical website, and online source study. One of the reasons for the difference in opinion between preclinical and clinical students could be that in clinical years' teaching, learning and assessment are patient-oriented and it cannot be learned by lecture handouts. Moreover, with the passage of time, they become accustomed to reading and understanding textbooks and realizing that without studying books better and a deep understanding of the subject, mastering the topic is not possible. Additionally, studying textbooks facilitates their chances of passing the Saudi Licensing Exam and fellowship exams as well. It may be explained in a way that by attaining maturity in the transition from the preclinical to the clinical phase and with the help of their seniors, the clinical group students gradually develop an interest in the habit of study [19].

In the Middle East, all the newly admitted medical students undergo training in English and pre-medical science subjects like biology, physics, and organic chemistry in the first year, called the foundation year [20]. In our study, we observed, through student’s feedback, that understanding written English remains the biggest hurdle for our students. Around 67% of the clinical group students complained of their poor understanding and frequent consultation of the dictionary to find out meanings. Almost a similar percentage of students committed their disliking for teaching material written in English. The pre-clinical group in whom this problem is of more intensity tried to ignore the question by avoiding a clear-cut response. This problem has also been observed in other studies in Saudi Arabia, and their recommendation was to introduce English teaching from the school level [21-22]. We suggest that the incorporation of an English language session at least once weekly in the first two preclinical years will help improve our students' understanding and speaking skills.

There is an apparent difference between the preclinical and clinical year students' approach to spare-time activities. It could be due to the difference in the burden of the studies, teaching and learning strategies, and assessment approaches. In the clinical years, it is more patients0oriented like short and long cases, clinical presentations, case-based learning, and objective structured clinical examinations. These differences compel clinical year students to adopt different approaches. In our study, we observed that social media software like Facebook and WhatsApp kept both groups of students busy most of the time, and it was especially marked in the clinical group, which can be explained as follows. In clinical training, the students prefer professional groups and most of the messages are sent via WhatsApp. This notion gets strengthened by the finding of a similar number of clinical student’s interest in an online medical literature study. Alwagait et al. mentioned both the negative and positive aspects of social media addiction in the younger generation of the Middle East and especially of Saudi Arabia [23]. According to recent studies, it was pointed out that KAU students are extensively using different social media [24-25].

The present study found that textbook reading and frequent visits to medical websites were consistently observed in the clinical group and the majority of successful preclinical students were taking class notes and reading the lecture handout provided by the teachers. Around 80% of preclinical students admitted that all the latest subject-specific books are available in the college library, and the teachers and study guides provided at the start of each module recommended the study of subject-specific books. Abdulghani et al. (2014) observed that the students who excel in the medical profession are frequently those who develop purposeful reading habits and are close observers of recent developments in medical fields [26].

Our results also show that students are more influenced and inclined toward available online resources. However, the problem is the selection of appropriate websites and tools from the available abundant websites. In this regard, the teacher can play an important role by guiding the students’ in choosing authentic tools and websites. Students are very eager, motivated, and dedicated; they need proper direction in their studies to excel in the field.

It is suggested that each department in the college should select the appropriate online tools and websites related to that course and provide the lists to the students in the study guide, so the students can take benefit from these websites. Furthermore, students must be encouraged to learn English from the school level so that they may not find the English language unfamiliar during their professional studies.

Limitations

There are certainly some limitations to our present study. The main limitation lies in being a single center, with a small sample size and a questionnaire-based study, so the chances of the student’s response biases are present. 

## Conclusions

Our results show that students in the clinical phase had a more methodical approach to professional studies and a difference in spare-time activities. The present seriousness of students towards their professional studies on exposure to the clinical environment is a very positive sign and needs to be encouraged.
